# Interactive Digital Health Tools to Engage Patients and Caregivers in Discharge Preparation: Implementation Study

**DOI:** 10.2196/15573

**Published:** 2020-04-28

**Authors:** Theresa E Fuller, Denise D Pong, Nicholas Piniella, Michael Pardo, Nathaniel Bessa, Catherine Yoon, Robert B Boxer, Jeffrey Lawrence Schnipper, Anuj K Dalal

**Affiliations:** 1 Brigham and Women's Hospital Boston, MA United States; 2 Harvard Medical School Boston, MA United States

**Keywords:** patient engagement, care transitions, health information technology, implementation science

## Abstract

**Background:**

Poor discharge preparation during hospitalization may lead to adverse events after discharge. Checklists and videos that systematically engage patients in preparing for discharge have the potential to improve safety, especially when integrated into clinician workflow via the electronic health record (EHR).

**Objective:**

This study aims to evaluate the implementation of a suite of digital health tools integrated with the EHR to engage hospitalized patients, caregivers, and their care team in preparing for discharge.

**Methods:**

We used the Reach, Effectiveness, Adoption, Implementation, and Maintenance (RE-AIM) framework to identify pertinent research questions related to implementation. We iteratively refined patient and clinician-facing intervention components using a participatory process involving end users and institutional stakeholders. The intervention was implemented at a large academic medical center from December 2017 to July 2018. Patients who agreed to participate were coached to watch a discharge video, complete a checklist assessing discharge readiness, and request postdischarge text messaging with a physician 24 to 48 hours before their expected discharge date, which was displayed via a patient portal and bedside display. Clinicians could view concerns reported by patients based on their checklist responses in real time via a safety dashboard integrated with the EHR and choose to open a secure messaging thread with the patient for up to 7 days after discharge. We used mixed methods to evaluate our implementation experience.

**Results:**

Of 752 patient admissions, 510 (67.8%) patients or caregivers participated: 416 (55.3%) watched the video and completed the checklist, and 94 (12.5%) completed the checklist alone. On average, 4.24 concerns were reported per each of the 510 checklist submissions, most commonly about medications (664/2164, 30.7%) and follow-up (656/2164, 30.3%). Of the 510 completed checklists, a member of the care team accessed the safety dashboard to view 210 (41.2%) patient-reported concerns. For 422 patient admissions where postdischarge messaging was available, 141 (33.4%) patients requested this service; of these, a physician initiated secure messaging for 3 (2.1%) discharges. Most patient survey participants perceived that the intervention promoted self-management and communication with their care team. Patient interview participants endorsed gaps in communication with their care team and thought that the video and checklist would be useful closer toward discharge. Clinicians participating in focus groups perceived the value for patients but suggested that low awareness and variable workflow regarding the intervention, lack of technical optimization, and inconsistent clinician leadership limited the use of clinician-facing components.

**Conclusions:**

A suite of EHR-integrated digital health tools to engage patients, caregivers, and clinicians in discharge preparation during hospitalization was feasible, acceptable, and valuable; however, important challenges were identified during implementation. We offer strategies to address implementation barriers and promote adoption of these tools.

**Trial Registration:**

ClinicalTrials.gov NCT03116074; https://clinicaltrials.gov/ct2/show/NCT03116074.

## Introduction

The transition from hospital to ambulatory care is a vulnerable time for patients and stressful for their caregivers: new treatments have been initiated, conditions require monitoring, and the plan is in flux. Approximately 19% to 28% of patients experience preventable adverse events after discharge, many due to suboptimal monitoring of conditions, medication errors or nonadherence, and failure to execute the recovery plan [1-6]. During hospitalization, discharge planning is often initiated late, and input from patients regarding their preparedness is frequently lacking, which may lead to delays and dissatisfaction [7]. After discharge, patients report problems related to follow-up, medications, and self-care; have unanswered questions that could have easily been addressed before discharge [8]; and often feel *more relieved than burdened* when readmitted [9]. Lack of patient engagement during the process of discharge preparation may contribute to avoidable adverse events and costly readmissions [10], particularly those that occur early after hospitalization [11,12].

To date, efforts to enhance and standardize discharge practices have typically targeted clinicians [13,14]; interventions directed at patients provide an opportunity to improve patient understanding, self-management, and postdischarge outcomes [15]. National agencies (eg, Agency for Healthcare Research and Quality [AHRQ], Centers for Medicare and Medicaid Services) are attempting to engage patients and caregivers more broadly by offering access to discharge preparation materials that include checklists for patients [16,17]. Few institutions have determined how best to operationalize these tools for patients. Digital health technology could be leveraged to more proactively engage patients, caregivers, and clinicians during the process of discharge preparation [18-21]; however, currently available patient-facing digital health tools such as patient portals have gaps in functionality with regard to assessing discharge readiness, are not well integrated with the electronic health record (EHR), and present challenges when used during hospitalization [18,21-23]. Although it is technically feasible to administer a discharge checklist through a patient portal or mobile device [24,25], hospitals lack knowledge about the potential for adoption and perceived utility of these tools for patients and clinicians in a real-world clinical setting, as well as potential barriers for sustaining the intervention from an organizational perspective.

To address this knowledge gap, we designed and developed an interactive patient-centered discharge toolkit (PDTK), a suite of EHR-integrated digital health tools that enabled patients to self-assess and communicate discharge preparedness to their care team and request secure text messaging with a hospital physician after discharge as part of a project funded by the AHRQ. Guided by the Reach, Effectiveness, Adoption, Implementation and Maintenance (RE-AIM) framework, we conducted a mixed methods study to describe the use, adoption, and perceived utility of the PDTK, as well as the key challenges encountered during implementation [26].

## Methods

### Overview

We used RE-AIM (Table 1), a framework designed to address issues related to the implementation of health services and clinical informatics research [26], to identify research questions to evaluate the feasibility and acceptability of the PDTK (Table 2) in a real-world clinical setting. Specifically, we employed a variety of quantitative and qualitative methods to assess the Reach and potential for Adoption, while identifying barriers to Implementation and strategies to Maintain the PDTK for hospitalized general medicine patients. Effectiveness of the intervention on outcomes will be evaluated in future studies.

**Table 1:** Reach, Effectiveness, Adoption, Implementation, and Maintenance framework: research questions and methods of analysis by dimension.

Dimension		Methods	Results
Reach	How many patients participate and why do they choose to decline?	Descriptive analysis of patients approached, and enrolled, including reasons for declining	Main results Table 3
	What types of patients use the patient-facing PDTK^a^ components?	Descriptive analysis of patient characteristics and hospitalization metrics from administrative databases, and whether they did or did not submit a checklist, or watch the video	Main results Table 3
Effectiveness	Does the PDTK activate patients at discharge?	Interviews at discharge to assess proportion of patients with Patient Activation Measure scores >55 (level 3 or 4)	Future study
	Will the PDTK favorably impact health care resource utilization after discharge?	Medical record review and phone interviews (30 days after discharge) to determine the proportion of patients with ≥1 unscheduled emergency department visit or readmission	Future study
	Can a checklist identify patients’ discharge concerns?	Descriptive analysis of patients' responses to checklist items	Main results Table 4
Adoption	How many clinicians participate, and what types of clinicians use the clinician-facing PDTK components?	Total number and percentage of clinicians of different types accessing the dashboard column and initiating postdischarge messaging	Table 4
Implementation	How frequently is each PDTK component utilized by patient and clinician participants?	Percentage of approached patients watching the video, completing checklist, and requesting postdischarge messaging Percentage of clinicians accessing dashboard column and initiating postdischarge messaging	Table 4
	Is the PDTK perceived to be valuable for patients and clinicians?	Descriptive analysis of survey results administered to patient participants Thematic analysis of content from semistructured interviews of patients and focus groups of clinicians	Patient survey results Table 5
Maintenance	What barriers, unintended consequences, and workflow challenges are encountered? What strategies are required to incorporate the PDTK into operations?	Thematic analysis of content from semistructured interviews of patients and focus groups of clinicians	Tables 5 and 6

^a^PDTK: patient-centered discharge toolkit.

### Setting and Participants

**Figure 1:**
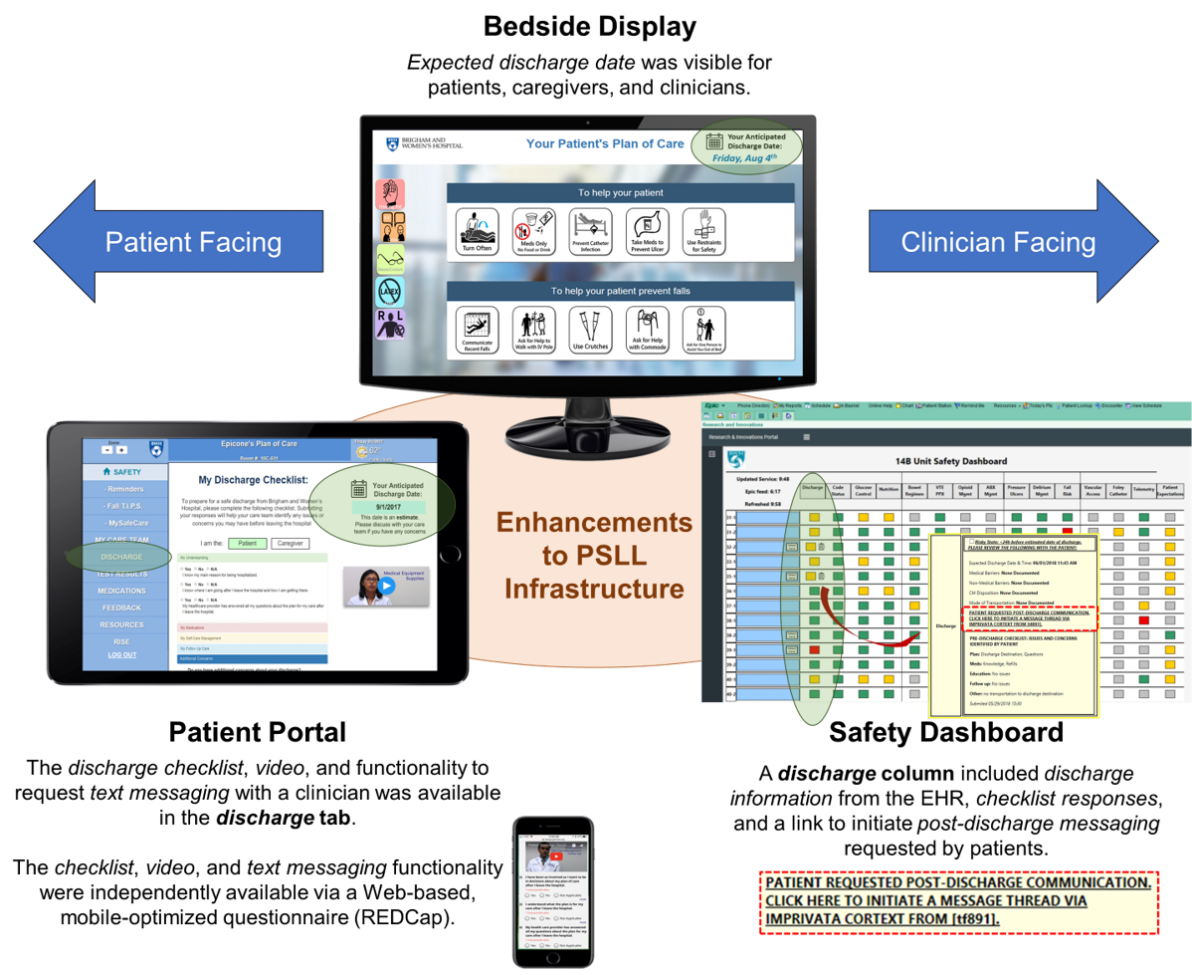
Patient-centered discharge toolkit: Enhancements to the EHR-integrated digital health infrastructure. PSLL: Patient Safety Learning Laboratory; EHR: electronic health record.

The PDTK study (Clinicaltrials.gov NCT03116074) was approved by the Partners’ institutional review board and was conducted at Brigham and Women’s Hospital in Boston, MA. The study was conducted on three 30-bed general medicine units from December 2017 through July 2018 in parallel with our AHRQ-funded Patient Safety Learning Laboratory (PSLL; Clinicaltrials.gov NCT02969343) reported elsewhere [27-30]. As part of the PSLL, we integrated a *bedside display* for patients and clinicians, a *patient portal* for patients and caregivers, and a *safety dashboard* for clinicians into our EHR environment (Epic Systems, Inc, Verona, WI) [29,30]. These applications used enterprise data services to obtain clinical data from the EHR in real time [19,30-33]. This EHR-integrated digital health infrastructure (Figure 1) served as a platform on which to incorporate enhancements for the independently funded PDTK study. The key focus of the PSLL was to use systems engineering and human factors methods to design, develop, and implement tools to prevent harm *in the hospital* (eg, falls, catheter-associated urinary tract infections) [29]. The goal of the PDTK study was to improve safety *during transitions out of the hospital* by designing, developing, and implementing enhancements to the PSLL infrastructure (Table 2) based on clinician end-user requirements and organizational priorities.

The PDTK comprised enhancements to each of the 3 components of the PSLL technical infrastructure: bedside display, patient portal, and safety dashboard (see Multimedia Appendix 1).

Each of the 3 study units was codirected by a physician and nurse pair and staffed by its own group of nurses. Patients admitted to each unit were cared for by 1 of 2 geographically localized general medicine teams comprising residents or physician assistants and a supervising hospitalist attending physician [34]. These medical teams rotated approximately every 2 weeks. A few off-service patients (ie, admitted to a service other than general medicine) were admitted to these units under the care of a different attending physician.

Any English- and Spanish-speaking patient admitted to these units under the general medicine service was eligible to participate by using any of the patient-facing PDTK components (checklist, video, and secure messaging). For patients who did not have the capacity to consent (as determined by a member of the care team), a caregiver (a designated health care proxy) could participate on their behalf. Patient or caregiver participants were offered access to an acute care patient portal on either personal devices or study-issued mobile devices (iPad Air, Apple, Inc, Cupertino, CA) as part of the concurrent PSLL study [19]. Any clinician (nurse, resident, physician assistant, and attending) caring for a general medicine patient admitted to these units was eligible to participate: all clinicians had access to EHR-integrated digital health infrastructure as part of the concurrent PSLL study and could therefore access the clinician-facing PDTK components (safety dashboard and secure messaging).

**Table 2:** Description of core components of the patient-centered discharge toolkit. Patient-facing Patient Safety Learning Laboratory (PSLL) tools: patient portal and bedside display; clinician-facing PSLL tools: bedside display and safety dashboard.

Component	Description
EDD^a^ display	Current EDD from the EHR^b^ was visible to patients on the patient portal and bedside display, and to clinicians on the bedside display and safety dashboard (Figure 1, green circles)
Discharge video	Patients could choose to watch a Web-based video of a clinician talking through each checklist item at an appropriate health literacy level Embedded via a hyperlink into the patient portal and REDCap (Research Electronic Data Capture, Nashville, TN) survey Available in English (clinician) and Spanish (medical interpreter)
Discharge checklist	A 16-item checklist that was available in English or Spanish could be completed by patient or caregiver via the patient portal or REDCap survey on a mobile device approximately 24 to 48 hours before EDD Dichotomous responses were sent to EHR-integrated safety dashboard in real time via API^c^
Clinician dashboard discharge column	Green flags identified patients with an EDD more than 1 day from the current date Yellow flags identified patients with an EDD less than 1 day from or equal to the current date Red flags identified patients with an EDD that was either not entered or past the current date; for patient portal enrollees, indicated that a checklist had not been completed when the current date was within 1 day of the EDD Checklist icon identified patients who had completed checklist and were awaiting clinician review *No* or *unsure* responses to checklist items were displayed by domain; free-text entries were displayed as additional patient-reported concerns; clinicians could address any unsatisfied item as needed (eg, unable to pay for medication and patient unaware of follow-up) Displayed key data from the EHR (medical and nonmedical barriers to discharge, discharge destination, and transportation) A link to initiate secure messaging was displayed for patients who requested postdischarge messaging
Secure messaging postdischarge	A secure messaging thread was opened by a clinician (opt-in process) via a link in safety dashboard (Figure 1, red dashed box) Patients were invited by their discharging clinician (attending, senior resident) to communicate up to 7 days on receiving an SMS text with a hyperlink to a mobile-optimized messaging portal Clinicians messaged with the patient via a HIPAA^d^-compliant app (Imprivata Cortext) on their mobile phone (without giving the patient access to their mobile phone number)

^a^EDD: expected discharge date.

^b^EHR: electronic health record.

^c^API: application programming interface.

^d^HIPAA: Health Insurance Portability and Accountability Act.

### Iterative Refinement of Intervention Components

In previous work, we engaged patient advisors, clinical stakeholders, information system professionals, and quality and safety leaders to identify gaps in discharge processes [19,35]. For this study, during the design and development phase, we conducted informal workflow observations on study units and interviews with stakeholders to identify end-user requirements for addressing these gaps by engaging patients and clinicians in discharge preparation while aligning with key organizational priorities: engaging patients to improve patient satisfaction, improving expected discharge date (EDD) documentation in the EHR, and reducing 30-day hospital readmissions [31]. For example, improving EDD accuracy—defined by our institution as the percent of final EDD entries equal to the actual discharge date (ADD)—was an organizational priority for improving operational throughput. Thus, to ensure timeliness of checklist completion and review of checklist responses by the care team, we enhanced the EHR-integrated *patient portal*, *bedside display*, and *safety dashboard* to improve the visibility of the EDD for both patients and clinicians (Figure 1). We presumed that the likelihood of checklist submission by patients and review by clinicians would be dependent on where patients were in their hospital course as well as their currently documented EDD.

**Figure 2:**
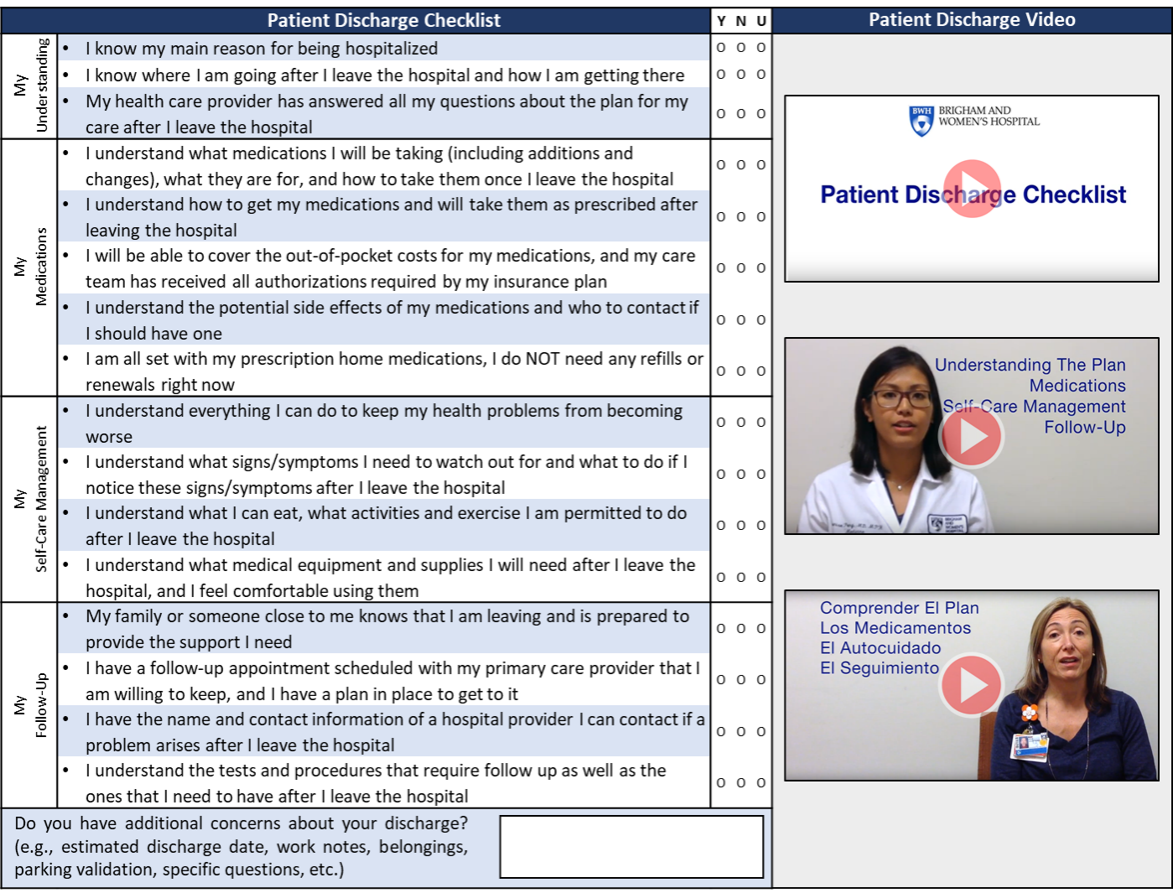
Discharge checklist and video.

As in the concurrent PSLL study [27,29,36], we applied user-centered design principles to refine patient- and clinician-facing intervention components (Figure 1) to ensure that we addressed end-user needs [31]. For the discharge checklist (Figure 2, left), our goal was to improve structure and organization, validate content, and clarify wording and utility. Key refinements were identified through multiple iterations of the original checklist within our research team (in part based on our experience with a transitions study funded by the Patient-Centered Outcomes Research Institute [37]), 2 sessions with our hospital’s patient and family advisory council, and a short pilot in which we administered a paper-based prototype to a convenience sample of 10 hospitalized patients and requested feedback. On the basis of the feedback from unit nurses and patient advisors, we also created a video to help hospitalized patients understand the purpose of completing the checklist to prepare for discharge. To develop the discharge video (Figure 2, right), we adapted a method previously demonstrated to improve patients’ understanding of their medical condition and care plan [38]. Finally, we determined that patients would need to watch the video and complete the checklist via one of several workflows: using the patient portal on a hospital-issued mobile device, using their own mobile device, or having research staff coach patients or caregivers to complete the checklist and then submit responses on their behalf.

**Figure 3:**
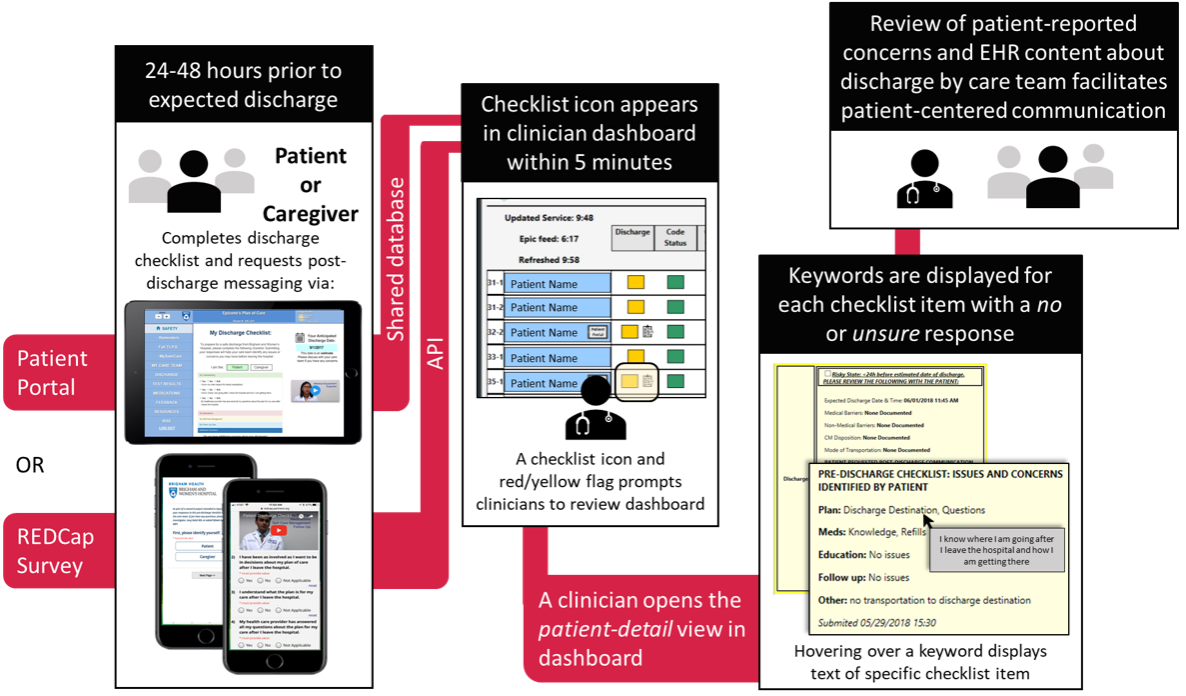
Checklist submission and review. EHR: electronic health record.

Next, we made discharge process–based enhancements to the PSLL technical infrastructure integrated with our EHR to ensure that patient-reported information from the checklist would be communicated to the care team as the EDD approached. On the basis of the feedback from patients and clinical unit leadership, we confirmed that patients would want their care team to have access to the checklist responses sufficiently before actual discharge to allow time for the care team to review and address any issues (see *Enrollment* below). Thus, we developed a checklist submission and review process (Figure 3) to ensure that checklist responses submitted by patients would be visible for clinicians to review in the EHR in real time.

The discharge checklist (Figure 2, left), originally created by Coleman [24], was adapted for our institution [4], and further refined into a four-domain, 16-item discharge checklist. Questions were simplified, reordered, and separated into four domains: *My Understanding*, *My Medications*, *My Self-Care Management*, and *My Follow-up*. Dichotomous checklist responses (*Yes*/*No*) were determined to be too strict (ie, patients were uncertain of their response and did not want to check yes or no); thus, a third option (*Unsure*) and a box for a free-text response was added. A caregiver version to be completed by health care proxies, as well as a Spanish version (approved by our hospital’s interpreter services) was created to make the checklist more inclusive of patients who lacked capacity or who did not speak English as their primary language (Multimedia Appendix 2).

The discharge video (Figure 2, right) was developed by creating an English version of a script in which a clinician guided the viewer through each checklist domain. This script was translated into Spanish and approved by our hospital’s interpreter services. A mobile device (iPad Air, Apple, Inc) was used to film English and Spanish versions. Video editing software (iMovie, Apple, Inc) was used to produce the videos. The videos were uploaded onto a video-hosting site (YouTube, LLC); hyperlinks to these videos were incorporated into the patient portal and REDCap (Research Electronic Data Capture, Nashville, TN; see Checklist Submission section).

### Checklist Submission

The two options available for patients to complete and submit the checklist were as follows: the patient portal or a Web-based REDCap survey [19,39]. REDCap submissions could be completed by patients on a mobile device (via a hyperlink emailed to the patient), or by study staff who would submit the responses on their behalf. Checklist responses submitted via the patient portal were made visible in the safety dashboard via a shared database. Checklist responses submitted via REDCap were routed to the safety dashboard using the REDCap application programming interface and matched to the corresponding patient using key identifiers (medical record number and admission date). In either case, checklist responses were displayed on the safety dashboard for clinicians within 5 min of submission.

### Checklist Review

A new discharge column in the safety dashboard displayed key data elements (medical and nonmedical barriers, EDD) from the EHR (Epic Systems, Inc, Verona, WI) using enterprise Web services [27]. EHR data were transformed into clinical decision support using color-coded *flags*: red=*action needed*; yellow=*risky state*; green=*guideline compliant*; gray=*not applicable*. For example, a red flag appeared if the current date was past the EDD, no EDD was entered, or the current date was within 24 hours of the EDD, but no checklist had been submitted. A checklist icon appeared on the safety dashboard on successful submission. Checklist items with a *no* or *unsure* response were briefly summarized as a keyword on the *patient-detail* view of the safety dashboard (eg, *Meds: Access/Adherence*) with a hover-over displaying the specific item answered by the patient (eg, *I understand how to get my medications and will take them as prescribed after I leave the hospital*). Patients were permitted to enter their mobile phone number during checklist submission to request secure messaging (Imprivata, Lexington, MA) with a hospital physician (attending or senior resident) for up to 7 days after discharge [19,27,40]. A link appeared in the safety dashboard for the clinician to initiate a message thread with the patient.

### Patient and Caregiver Enrollment

The intervention went live in December 2017, starting with a 1-month wash-in period in which we debugged various technical components. During business days, recruitment lists were created in which the care unit and patient approach order were randomized to minimize confounding (eg, always starting enrollment on the top floor of the hospital and ending at the bottom). All patients admitted to intervention units for at least 24 hours with EDDs within 24-48 hours were eligible. Research assistants asked the nurses caring for these patients if they were appropriate to approach, temporarily excluding any patient who was nonverbal, incapacitated, or behaviorally not safe. When an eligible patient was confirmed as not capable of participating, the research assistant attempted to identify a caregiver (a designated health care proxy). Patients and caregivers who were not available for recruitment (eg, off unit getting a test) were reapproached later that day or on subsequent days if they still met eligibility criteria. Research assistants then asked eligible patients (or caregivers) to watch the video and complete the checklist (including entering their mobile phone number to request postdischarge messaging) via the patient portal or REDCap workflow. A patient was considered enrolled on successful submission of the checklist.

### Measurements and Data Collection

We used mixed methods to evaluate our implementation experience per each RE-AIM dimension (Table 1). Specifically, we used quantitative methods to measure usage and perceived utility of intervention components (Reach, Adoption, and Implementation). We used qualitative methods to assess barriers to and facilitators of Implementation and Maintenance from the patient and clinician perspective.

#### Usage of Intervention Components (Quantitative)

We captured the number of times the video was watched, the checklist was submitted, and postdischarge messaging was requested by patients. We captured the number and type of patient-reported concerns for each checklist submitted, as well as the number of times clinicians accessed the discharge column on the safety dashboard to view patient-reported concerns and click on the link to initiate postdischarge messaging.

#### Patient Surveys (Quantitative)

We previously reported *usability* of the patient portal, which included a discharge module with an earlier, noninteractive version of the checklist [19]. To better understand *perceived utility* of the PDTK, we asked a convenience sample of enrolled patients to participate in a survey guiding them through each intervention component. The 2-part survey, based on prior work [32], asked participants to rate their perceived readiness for discharge and willingness to self-assess discharge preparedness on a 5-point Likert scale. After watching the video, completing the checklist, and viewing how clinicians could visualize checklist responses in the safety dashboard, participants rated statements about their confidence that the intervention would facilitate self-identification and communication of discharge concerns to their care team.

#### Patient Interviews (Qualitative)

We verbally consented and conducted semistructured interviews with a convenience sample of English-speaking patients within 24-48 hours of anticipated discharge. Care team members (nurse, physician assistant, and resident or attending) were asked to identify patients within this time frame who were present in their rooms and would be amenable to participating in a brief interview. To reduce sampling bias, potential participants were selected to create a diverse sample based on age, gender, and reason for hospitalization. To minimize selection bias, patients were assured that their participation would not influence their care team’s medical decisions, and care teams were not told which patients agreed to participate. All participants completed the checklist and watched the video either before or at the time of the interview. Study staff (DP and NP) trained in qualitative research methods conducted the interviews using a semistructured interview guide that explored (1) patient experiences completing the checklist and viewing the video, (2) addressal of discharge concerns by the care team, and (3) pros and cons of using these tools. Interviews were digitally recorded, transcribed, and reviewed for accuracy, and conducted until thematic saturation was achieved [41].

### Clinician Focus Groups (Qualitative)

After the study was completed, we conducted focus groups with physicians, physician assistants, and nurses to assess implementation barriers and facilitators until thematic saturation. Using a structured guide, we asked about EDD entry via the EHR, perception of the checklist workflow, usage of the discharge column of the safety dashboard to review checklist responses and initiate postdischarge messaging, and awareness of patient-facing components (bedside display and patient portal). Focus group discussions were digitally recorded, transcribed, and reviewed for accuracy.

### Statistical and Qualitative Analyses

Descriptive statistics were used to report patient demographic and administrative data, quantify patient-reported concerns, calculate the frequency of tool use by patients and clinicians, and quantify survey data. We calculated the proportion of patients (or caregivers) completing the checklist via the patient portal or REDCap. All qualitative data collected from patient and clinician participants were transcribed, openly coded, and analyzed using the constant comparative method [42]. Two researchers (DP and NP) independently coded all transcripts line by line using Word (Microsoft, Inc, Redmond, WA). The code structure was revised as needed to capture novel concepts, adapt, and merge existing concepts; transcripts were coded with each iteration of the codebook, with any discrepancies resolved during consensus meetings. This process was repeated until no novel concepts were identified, at which point, the 2 researchers again independently applied the final code structure to all transcripts. Key themes were identified in a final group consensus meeting with the study staff. Key implementation barriers were identified by study staff via a group consensus approach based on quantitative and qualitative data.

## Results

Of 752 patient-admissions, the patient (or caregiver) watched the video and completed the checklist in 416 (55.3%), and the patient/caregiver completed the checklist alone in 94 (12.5%). Research assistants made 313 attempts at approaching patients in the remaining 242 patient-admissions; however, the patient was unavailable (126), not appropriate per nurse (97), declined to participate (41), did not speak English or Spanish and no caregiver was available (33), did not respond by email when reminded (8), or encountered technical issues (8). The demographic characteristics of the 67.8% (510/752) patient-admissions (480 unique patients) in which a checklist was submitted and the 32.2% (242/752) patient-admissions (238 unique patients) in which the checklist was not submitted are reported in Table 3. In general, those who did not submit a checklist were older, more often Hispanic and non-English speaking, less often privately insured, had higher diagnosis-related group (DRG) weights and longer lengths of stay, and were typically discharged to a destination other than home.

**Table 3:** Demographics of patient admissions (N=752).

Characteristics			Submitted checklist (n=510)		Did not submit checklist (n=242)		*P* value
**Unique patients**			**480**		**238**		**—^a^**
	1 hospitalization	453		234			
	2 or more hospitalizations	27		4			
**Age (years), mean (SD)**			**58.6 (17.9)**		**62.4 (18.6)**		**.008^b^**
	Missing	—		14		—	
**Gender, n (%)**						**.94^c^**	
	Female	280 (54.9)		126 (52.1)			
	Missing	—		14 (5.8)			
**Race, n (%)**						**.13^c^**	
	White	340 (66.7)		140 (57.9)			
	Nonwhite	162 (31.7)		86 (35.5)			
	Missing	8 (1.6)		16 (6.6)			
**Ethnicity, n (%)**						**<.001**	
	Non-Hispanic	468 (91.8)		189 (78.1)			
	Hispanic	33 (6.5)		36 (14.9)			
	Unavailable	9 (1.7)		3 (1.2)			
	Missing	—		14 (5.8)			
**Primary language, n (%)**						**<.001^c^**	
	English	492 (96.5)		187 (77.3)			
	Non-English	11 (2.2)		40 (16.5)			
	Missing	7 (1.4)		15 (6.2)			
**Median income by ZIP code**						**.46^c^**	
	≤US $47,000	96 (18.8)		49 (20.3)			
	US $47,001 to US $63,000	124 (24.3)		48 (19.8)			
	Greater than US $63,000	272 (53.3)		131 (54.1)			
	Missing	18 (3.5)		14 (5.8)			
**Insurance status, n (%)**						**<.001^c^**	
	Private	195 (38.2)		70 (28.9)			
	Public (Medicaid, Medicare)	305 (59.8)		134 (55.4)			
	Other^c^	10 (2.0)		18 (7.4)			
	Missing	—		20 (8.3)			
**Primary care physician, n (%)**						**.01^c^**	
	In-network	229 (44.9)		125 (51.7)			
	Nonnetwork	280 (54.9)		103 (42.5)			
	Missing	1 (0.2)		14 (5.8)			
**Elix number of comorbidities, mean (SD)**			**4.22 (2.41)**		**4.53 (2.45)**		**.15^b^**
	Missing	—		14			
**Elix index, comorbidities, n (%)**						**.03^c^**	
	Less or = 0	104 (20.4)		33 (13.6)			
	1 to 5	96 (18.8)		29 (12.0)			
	6 to 10	94 (18.4)		26 (10.7)			
	11 or more	216 (42.4)		106 (43.8)			
	Missing	—		48 (19.8)			
**DRG^d^ weight, mean (SD)**			**1.83 (1.97)**		**2.26 (2.10)**		**<.001^b^**
	Missing	10		19			
**Length of stay, mean (SD)**			**8.78 (7.93)**		**11.5 (13.7)**		**.02^b^**
	Missing	—		14			
**Discharge destination, n (%)**						**.003^c^**	
	Home	410 (80.4)		133 (55.0)			
	Facility	92 (18.0)		58 (24.0)			
	Other	7 (1.4)		2 (0.8)			
	Missing	1 (0.2)		49 (20.3)			
**Readmissions within 30 days, n (%)**						**.18^c^**	
	Yes	88 (17.3)		39 (16.1)			
	No	422 (82.7)		140 (57.9)			
	Missing	—		63 (26.0)			

^a^Not applicable.

^b^*P* value calculated by Wilcoxon test.

^c^*P* value calculated via chi-square test.

^d^Nonstandard insurance or self-insured.

^e^DRG: diagnosis-related group.

Usage of each PDTK component for the 510 patient-admissions in which a checklist was submitted is reported in Table 4. Although the patient was enrolled in the acute care patient portal in 173 (33.9%) of the 510 patient-admissions, the patient portal was used to submit the checklist in 53 (10.4%); the remainder were submitted via REDCap. The checklist was submitted once and 2 or more times in 492 and 18 patient-admissions, respectively. The median (IQR_25,75_) days from initial checklist submission to EDD and ADD were 1 (1,2) and 2 (1,5), respectively. On average, 4.24 concerns were reported for each checklist submitted: the most commonly entered concerns by patients were about medications (664/2164, 30.68%) and follow-up (656/2164, 30.31%). The EDD was accurate in 307 (60.2%) of the 510 patient-admissions.

Of the 20 patient experience survey participants, 13 (65%) felt well prepared for discharge, and 16 (80%) stated that they would be willing to self-assess discharge preparedness via a checklist. After viewing how the PDTK components functioned, all (100%) completed the checklist, reporting an average of 5.1 concerns (17 understanding of the plan, 30 medications, 21 self-care management, and 34 follow-up); and 7 (35%) requested secure messaging after discharge; 13 (65%) felt that the checklist facilitated self-identification of potential issues before discharge; 15 (75%) believed that their care team would become aware of these issues via the safety dashboard; 10 (50%) felt more confident about what to do to prevent issues after leaving, and 15 (75%) felt confident that they could quickly communicate with a hospital physician via secure messaging should an issue arise postdischarge.

Of the 20 patients approached for semistructured interviews, 12 participated: 7 (58%) were male, 10 (83%) were white, the median age was 70.5 years, and 8 had public insurance. The most common reason for declining to participate was feeling unwell or tired. We identified two overarching themes about discharge preparation: (1) gaps in communication between patients and their care team resulting in patients feeling inadequately informed about their discharge care plan (eg, *I wasn’t informed and kept up to date with what was happening and the reason why. I understood on my own basically what was needed because I’ve gone through this before, but if it was the first time, I think I would have been very confused*); and (2) despite perceived communication gaps, patients were confident that their care team would address all of their questions and concerns before discharge (eg, *I knew everything was going to be done and things were going to be taken care of, but really, I didn’t feel informed, I really didn’t*). We also identified key themes regarding patient experiences using the checklist and video components of the PDTK (Table 5).

In total, 22 clinicians (8 physicians, 6 physician assistants, 8 nurses; mean age 36.9 years; 14 (14/22, 64%) female) participated in 1 of 3 focus groups from which we identified 3 major themes regarding the safety dashboard component of the PDTK (Table 5): low awareness and variable workflow, lack of optimization, and inconsistent leadership.

**Table 4:** Usage of patient-centered discharge toolkit component during 510 patient-admissions (480 unique patients).

Metric		Statistic	Comment
Discharge video watches, n (%)		416 (81.6)	Watched before checklist completion, most often the English version
**Discharge checklist version submitted, n (%)**			
	Patient	497 (97.5)	—^a^
	Caregiver	13 (2.5)	Consented if patient preferred or did not have capacity
**Electronic workflow used to submit checklist, n (%)**			
	Web-based REDCap survey	457 (89.6)	Submitted via a mobile device
	Patient portal (discharge module)	53 (10.4)	Could submit the checklist via the portal or REDCap
**Total number of concerns reported ^b^by domain**		**164**	**Most frequent items checked *no* or *unsure* by domain**
	Understanding the plan, n (%)	355 (16.4)	Understanding the main reason for hospitalization
	Medications, n (%)	664 (30.7)	Understanding changes to the medication regimen and how to get and take medications
	Self-care, n (%)	437 (20.2)	Understanding *red flag* signs and symptoms
	Follow-up, n (%)	656 (30.3)	Time and date of appointments, how to get to them
	Other, n (%)	52 (2.4)	Unaddressed clinical concerns, nonmedical barriers
**Safety dashboard discharge column**			
	Viewed by clinical staff during patient-admission, n (%)	210 (41.2)	Accessed safety dashboard’s patient-detail view or clicked acknowledgment check-box
	Total number of times accessed, n	631	Median (IQR 25,75): 2 (1,4) per patient-admission
	RN, n (%)	399 (63.2)	Unit-based bedside nurses
	MD, n (%)	180 (28.5)	Attending or resident
	Administrative, n (%)	44 (7.0)	Unit clerk
	Physician assistant, n (%)	8 (1.3)	Worked on separate nonresident service with attendings
**Secure postdischarge messaging (n=422^c^)**			
	Requested by patient, n (%)	141 (33.4)	Patient must have had mobile phone with a mobile web-browser
	Initiated by physician, n (%)	3 (2.1)	2 attendings, 1 senior resident

^a^Not applicable.

^b^A discharge checklist item for which the response was *no* or *unsure* was considered a patient-reported concern.

^c^Denominator reflects number of patient admissions in which postdischarge messaging was available.

**Table 5:** Key themes from patient interviews and clinician focus groups about patient-centered discharge toolkit components.

Theme		Description	Quote
**Checklist and video**			
	Valuable for patients	The checklist and video increased understanding of self-care needs and follow-up plans and promoted patient engagement and empowerment in the discharge process.	“I may think of questions I didn’t really have. Definitely worth it. It actually makes you think.” [Patient] “[The checklist] made the patient feel like a more active participant [in] their care…” [Clinician]
	Patient utility dependent on the timing of administration	The checklist and video were most useful when administered close to discharge but before a detailed discussion of discharge preparation by a care team member.	“Well, it was a little unclear given that we’re not about to leave. It’s hard to report on the process because it hasn’t actually happened yet.” [Patient]
**Safety dashboard**			
	Low awareness, variable workflow	Although clinicians were generally aware, checklist answers were variably viewed on the safety dashboard. Reinforcement and reminders to use the safety dashboard to review patient-reported discharge concerns were variable. The workflow for entering and updating EDD^a^ was inconsistent and included both clinical and nonclinical staff.	“[Discharge checklist responses] on the dashboard?... Did not know that.” [Clinician] “When it first rolled out there was a lot of information about it and then it just dropped off, and then the usage dropped off…” [Clinician] “[EDD] not really my workflow…I mean we’ll put in [the EDD], and it’ll get changed by a unit coordinator on a different pod.” [Clinician]
	Lack of optimization	Discharge column flag logic was often misinterpreted by different clinicians. Summarized checklist responses displayed in safety dashboard were too broad and nonspecific. Clinicians could not quickly access the entire checklist.	“The senior resident did not know really, what green [dashboard flags] meant...are [the patients] ready to be discharged?” [Clinician] “I would look at [the safety dashboard] sometimes and wonder what [the patient] clicked off [on the checklist], but sometimes I couldn’t tell exactly what they had questions about.” [Clinician]
	Inconsistent leadership	Usage was dependent on senior-level clinician leadership (attending or senior resident).	“…when the attendings were into it we were all into it for that week.” [Clinician]

^a^EDD: expected discharge date.

## Discussion

### Principal Findings

We used the RE-AIM framework to evaluate the feasibility and acceptability of a suite of EHR-integrated digital health tools to engage patients, caregivers, and clinicians in discharge preparation. Most patients agreed to watch the discharge video and complete the checklist to self-assess discharge preparedness when coached, and we did not encounter significant technical difficulty in our approach. The patient-facing tools were perceived to be valuable by both patients and clinicians, and most patient-reported concerns submitted via the checklist related to medications and follow-up. Clinician use of the safety dashboard discharge column to view these concerns was modest, mostly due to workflow challenges. A large percentage of patients requested postdischarge messaging, but very few clinicians opted in. Themes identified from our qualitative analysis suggest that timing of administration, additional workflow integration, optimization, and leadership are necessary to promote a more robust adoption of these tools.

We attribute the high rate of patient participation to flexible Web-based workflows and facilitation by research assistants. First, the iterative process to develop and refine the checklist and video incorporated feedback from patient advisers and institutional stakeholders, resulting in a product that was relevant and understandable to patients. Next, research assistants functioned as discharge advocates for study participants, guiding them through the process of viewing the video, and completing the checklist. This encouraged participation from patients who might otherwise have not been comfortable or motivated to do so independently. In addition, we included a video component and incorporated a checklist submission process that did not depend on the acute care patient portal: REDCap’s Web-based workflow was useful as a mobile app prototype and also circumvented key barriers to patient portal enrollment and use during hospitalization, such as patients’ dislike of having a separate log-in for the acute care patient portal [19,36]. In contrast, the direct hyperlink to the discharge checklist in REDCap offered more streamlined access (eg, no log-in was needed) for patients, and facilitation by research staff mitigated the perceived burden of submitting the checklist electronically on their own.

Although most patient participants perceived that the intervention would facilitate communication regarding discharge concerns to their care team, often this did not occur because of low uptake of the intervention by clinicians; we attribute this to low awareness of the intervention, inconsistent understanding of its purpose and how to use it, and lack of specificity of patient-reported concerns viewable on the safety dashboard. Specifically, although the safety dashboard discharge column logic was vetted by institutional stakeholders to align with hospital priorities (improving EDD accuracy), clinicians often misinterpreted safety dashboard flag colors (eg, green did not signify *safe for discharge*) and had a different understanding of responsibility for updating the EDD in the EHR because of inconsistent processes. In addition, if clinicians did not access the detailed view for the patient or click on the flag, they did not see the full text of the flag and what it meant, only its color [43]. Finally, variable use of the discharge column by physicians likely led to poor awareness of the link to initiate a secure messaging thread when requested by patients, and many were resistant to using this feature altogether.

As one of the first reported attempts at engaging patients, caregivers, and clinicians in discharge preparation using a suite of EHR-integrated digital health tools, findings from our implementation study, guided by the RE-AIM framework, offer several instructive lessons (Table 6). First, the optimal timing of when to complete the checklist is paramount. If completed too early, patients perceive less utility, anticipating that the care team would eventually address their concerns. If completed too late, concerns identified from the patients’ perspective are less likely to be communicated to clinicians, leading to potential deficiencies or delays in addressing them. Second, dashboard flag changes were not linked to relevant EHR data elements because of competing workflows (eg, a newly created process for documenting and escalating discharge barriers to hospital leadership) and technical limitations (eg, lack of a Web service to retrieve readmission risk scores from the EHR). Rather than linking flag changes to EDD documentation and timing, using the safety dashboard to review patient-reported concerns about discharge might be more clinically meaningful in the context of patient-specific readmission risk scores; this would be more consistent with the overall intent of the safety dashboard as a tool for proactively identifying patients at risk for harm [27,44], Third, improving EDD accuracy via this type of intervention is more likely to be achieved if the responsibility for updating EDD resides with clinicians rather than unit clerks and is clear to all parties. Patients and clinicians will then have more confidence in the EDD displayed on the patient portal, bedside display, and safety dashboard. Still, it is noteworthy that EDD accuracy for enrolled patients (307/510, 60.2%) was marginally higher than the EDD accuracy rate for general medicine (965/1702, 56.70%) as a whole. Finally, secure messaging after discharge clearly requires a better understanding of factors predicting whether clinicians will use this feature and how to incentivize its use.

**Table 6:** Implementation barriers and strategies to promote adoption.

Implementation barriers			Strategies to promote adoption
**Discharge video**			
	Timing and access of video after admission to the unit	Make videos available via the patient portal, bedside display, and television Engage nurses to have patients watch videos as EDD^a^ approaches	
	Too generic and impersonal	Have clinical unit leaders create unit-specific videos Create videos for each attending, play video for patient’s current attending by linking to the treatment team in the EHR^b^ Translate videos into common languages (eg, Spanish) using medical interpreters	
**Discharge checklist**			
	Timing and administration	Determine optimal timing of checklist administration for specific patient categories (eg, admissions for acute on chronic disease exacerbations, awaiting procedures, undifferentiated diagnoses) Demonstrate impact on key hospital priorities and process metrics (EDD accuracy, early hospital discharges)	
	Patients’ belief that clinicians will address all items	Encourage patients to review and update the checklist during their hospitalization Allow patients to update checklist responses as EDD approaches or changes	
	Checklist responses out-of-date owing to discharge delays	Identify workflow to update checklist after initial submission (eg, notification via the patient portal, email, or mobile app)	
**Dashboard discharge column**			
	Variable EHR data entry of key data elements (EDD, medical, nonmedical barriers)	Demonstrate how EDD can be viewed by patients (patient portal, bedside display) and clinicians (bedside display, dashboard) Add a confidence indicator that estimates the likelihood that EDD will equal ADD^c^ to manage patient and clinician expectations Demonstrate the value of structured EHR data entry for driving dashboard logic (flagging red when EDD not entered) Encourage checklist completion for patients at high risk for readmission by incorporating patient-specific readmission risk scores from EHR into logic Display barriers to discharge on the dashboard	
	Competing QI^d^ interventions	Understand current institutional priorities and emerging workflows for identifying and escalating discharge barriers Propose enhancements based on lessons learned from concurrent QI efforts to explain how the use of a checklist can prepare patients for postdischarge care (increasing patient satisfaction, reducing readmission rates) while maintaining or reducing the length of stay (by proactively identifying and overcoming barriers to timely discharge)	
	Poor specificity of patient-reported concerns viewed in the dashboard	Provide a link to discharge checklist questions and patient’s responses Link patient-reported concerns to specific clinical actions (eg, if poor understanding of the main diagnosis, update after visit summary with condition-specific educational materials)	
**Secure postdischarge messaging**			
	Physician resistance	Frame the initiation of secure messaging thread as an opt-in process Align with value-based incentives for clinical services (readmissions) Communicate success stories from early adopters to assuage fears (eg, excessive text messages from patients)	
	Managing patient expectations about whether physicians will initiate secure messaging	Educate patients about the opt-in process for attendings Encourage patients to request attendings to use this feature for clearly defined reasons (eg, concern about obtaining a key medication)	

^a^EDD: expected discharge date.

^b^EHR: electronic health record.

^c^ADD: actual discharge date.

^d^QI: quality improvement

### Limitations

Our study has several limitations. First, it was conducted for general medicine patients at a single institution without a control group; therefore, we could not evaluate the impact of our intervention on clinical outcomes. Similarly, our qualitative analyses were performed on convenience samples of participants, which may limit the generalizability of our findings. Second, we used research assistants to coach patients in submitting the checklist; although dedicated discharge advocates are becoming increasingly common, many institutions lack sufficient personnel [45]. In most hospitals, nursing staff could serve patients in this capacity as they are often the first to identify concerns reported by patients preparing for discharge. Third, we identified disparities among those who submitted and those who did not submit a checklist. Clearly, additional work is needed to address disparities in underrepresented groups to fully evaluate the utility of this intervention in a broader population. Finally, this was a hospital-centric intervention—we did not engage primary care physicians. Although our efforts at postdischarge messaging attempted to bridge the transition from inpatient to ambulatory care, the secure messaging vendor used in this study did not offer the ability to communicate with multiple care team members simultaneously, as we previously described [40]. Nonetheless, seamless communication with key ambulatory clinicians is important during the immediate postdischarge period [46,47].

### Conclusions

We believe that EHR-integrated digital health tools such as those we described will become increasingly useful as part of an institutional strategy to engage patients, caregivers, and clinicians in improving discharge safety if they simultaneously address key hospital priorities (eg, improving EDD accuracy and mitigating readmission risk). Currently, we are making further enhancements to the intervention components and their implementation. For example, we are reconfiguring the dashboard discharge column logic to more clearly identify patients at high risk for readmission, which should provide context for the types of concerns patients report after completing the checklist. Exploratory features, such as secure messaging with patients, clearly require further investigation to better characterize patient and clinician perceptions of its value and appropriate use after discharge. However, we believe that many of these features will become increasingly utilized to comply with new regulations (eg, Caregiver Advise, Record, Enable [CARE] Act) [48]. Finally, we plan to conduct rigorously designed studies to evaluate the impact of the PDTK on key outcomes during transitions, such as patient activation at discharge, postdischarge health care resource utilization, and hospital readmissions.

## Appendix

Multimedia Appendix 1 All patient safety learning laboratory enhancements. Enlarged images for viewing the details of the technological enhancements.

Multimedia Appendix 2 All checklist versions. Three versions of the discharge preparedness checklist were created (English speaking patients and caregivers, as well as Spanish speaking patients), listed in this appendix in that order.

## Abbreviations

ADD: actual discharge date
AHRQ: Agency for Healthcare Research and Quality
DRG: diagnosis-related group
EHR: electronic health record
PDTK: patient-centered discharge toolkit
PSLL: Patient Safety Learning Laboratory
RE-AIM: Reach, Effectiveness, Adoption, Implementation, and Maintenance
