# Supplementary figures and images for: Interactive Digital Health Tools to Engage Patients and Caregivers in Discharge Preparation: Implementation Study
Source: J Med Internet Res. 2020 Apr 28;22(4):e15573. doi: 10.2196/15573 (PMC7218608; doi:10.2196/15573)

## Slide 1
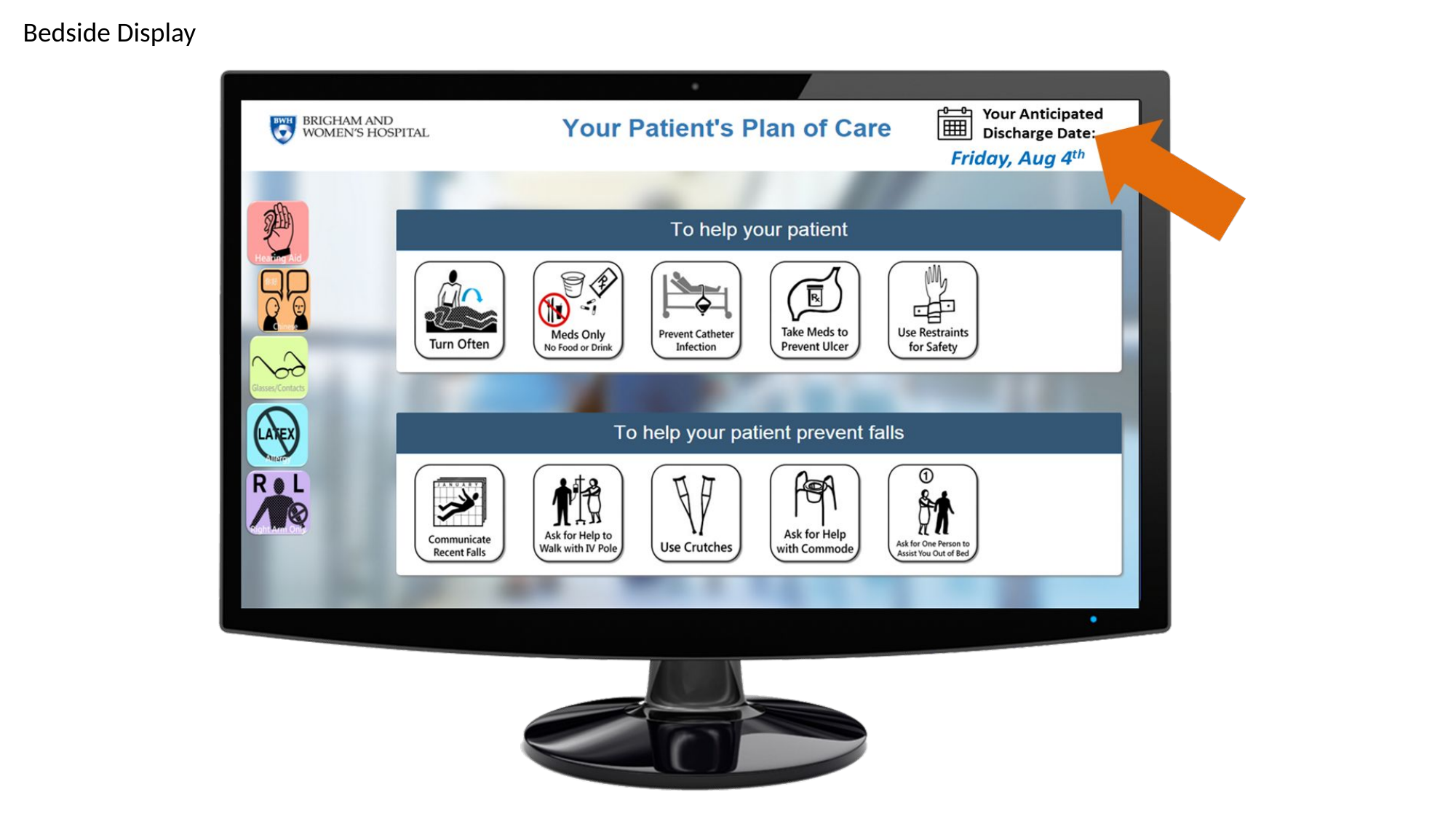

Bedside Display

## Slide 2
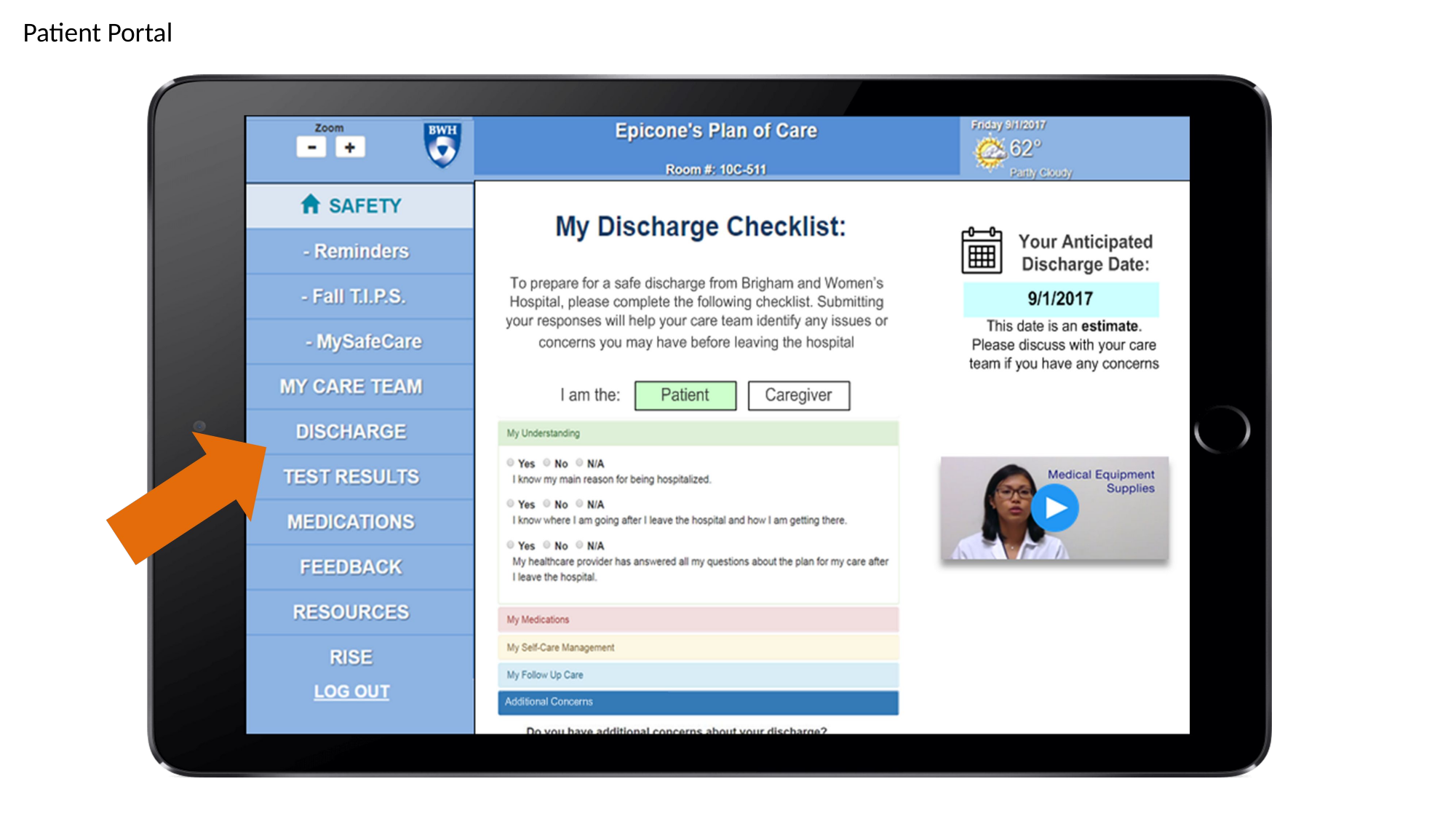

Patient Portal

## Slide 3
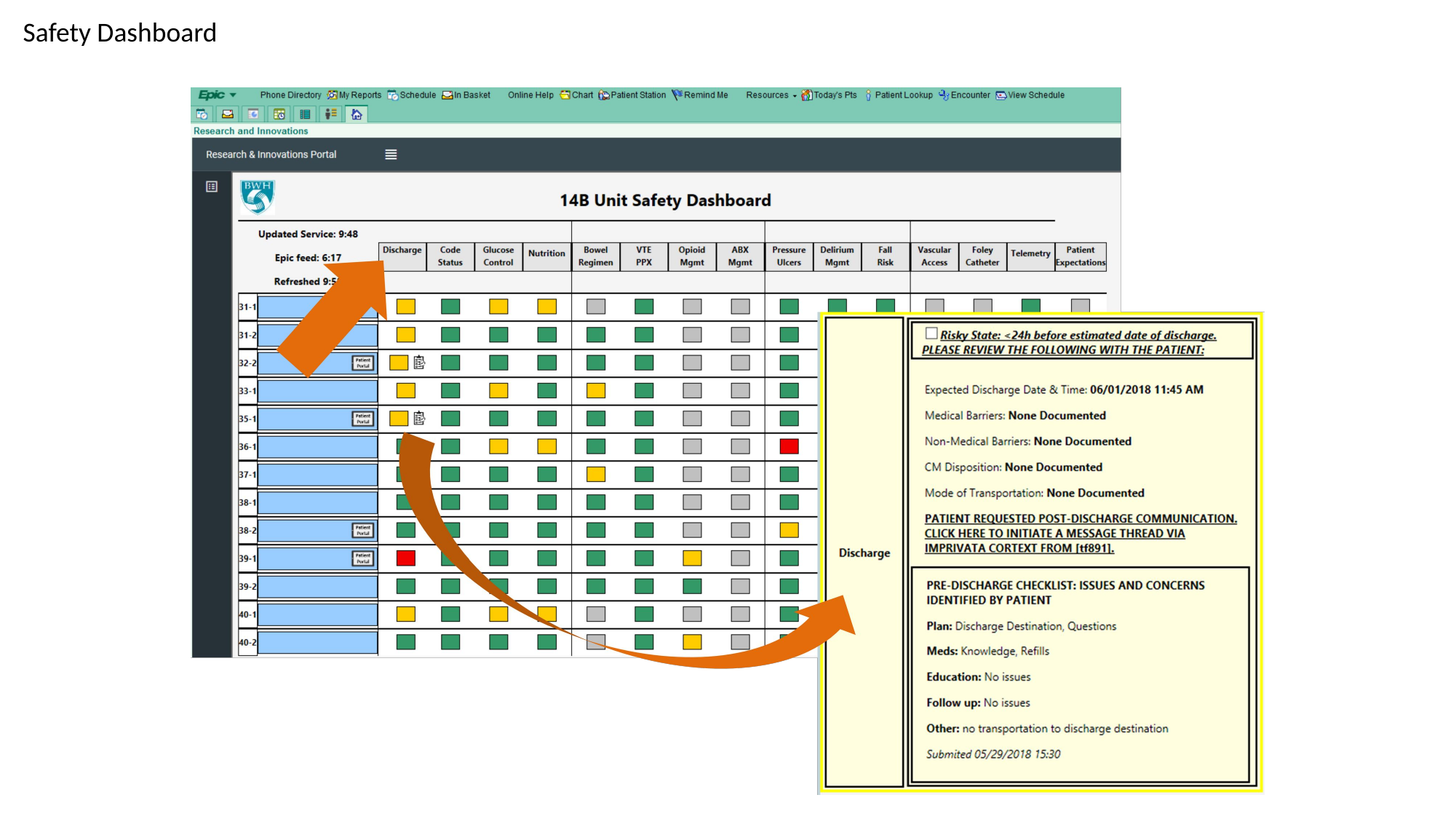

Safety Dashboard

## Slide 4
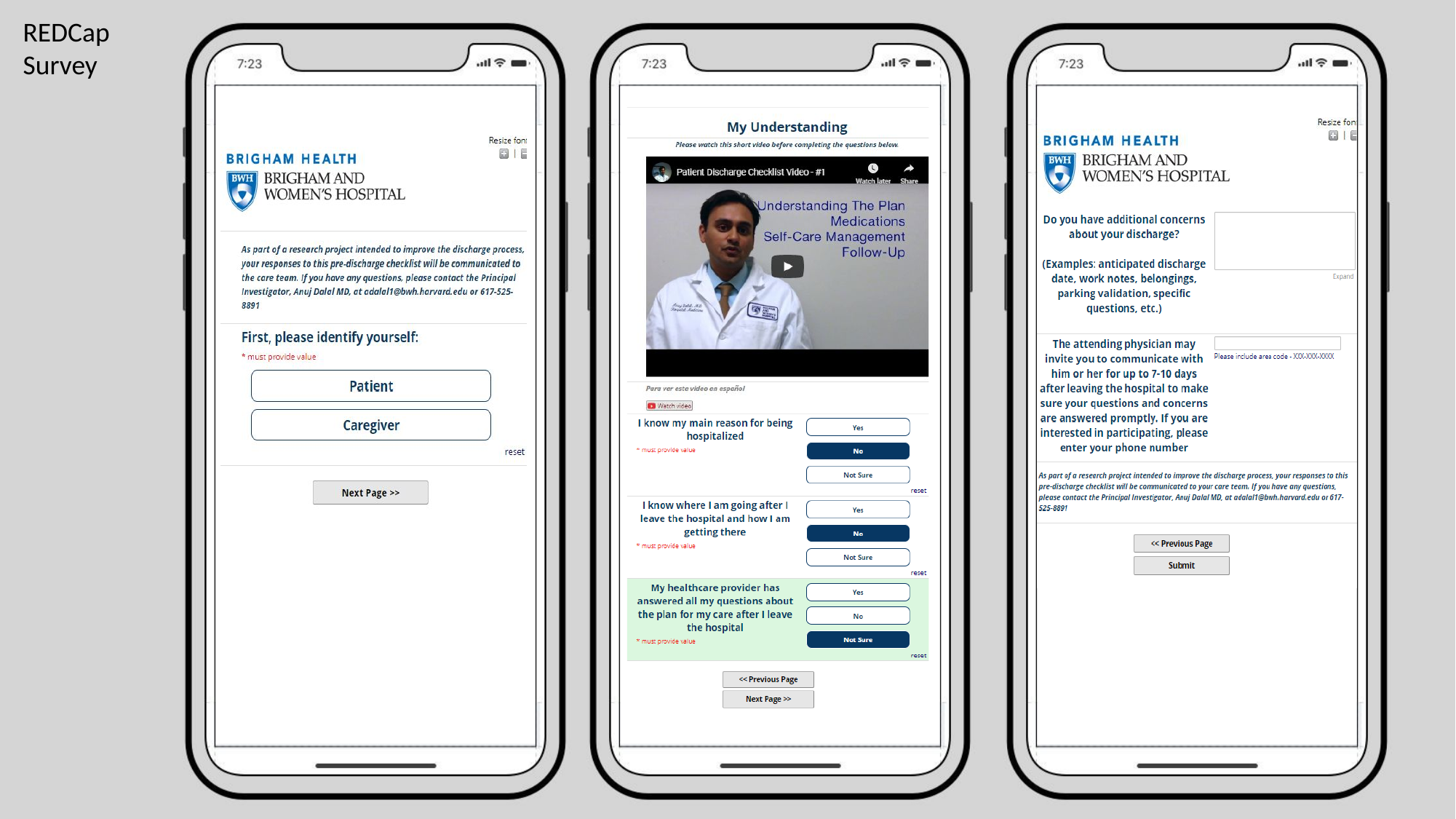

REDCap
Survey

Supplement: Multimedia Appendix 1 [file jmir_v22i4e15573_app1.pptx]
