# Supplementary material for: Interactive Digital Health Tools to Engage Patients and Caregivers in Discharge Preparation: Implementation Study
Source: J Med Internet Res. 2020 Apr 28;22(4):e15573. doi: 10.2196/15573 (PMC7218608; doi:10.2196/15573)

**Patient Discharge Preparation Checklist**

| **My Understanding** | Yes | No | Not Sure |
| --- | --- | --- | --- |
| I know my main reason for being hospitalized | € | € | € |
| I know where I am going after I leave the hospital and how I am getting there | € | € | € |
| My healthcare provider has answered all my questions about the plan for my care after I leave the hospital. | € | € | € |
| **My Medications** | Yes | No | Not Sure |
| I understand what medications I will be taking (including additions and changes), what they are for, and how to take them once I leave the hospital | € | € | € |
| I understand how to get my medications and will take them as prescribed after I leave the hospital | € | € | € |
| I will be able to cover the out-of-pocket costs for my medications, and my care team has received all authorizations required by my insurance plan | € | € | € |
| I understand the potential side effects of my medications and who I should contact if I have one | € | € | € |
| I am all set with my prescription home medications, I do **NOT** need any refills or renewals right now | € | € | € |
| **My Self-Care Management** | Yes | No | Not Sure |
| I understand everything I can do to keep my health problems from becoming worse | € | € | € |
| I understand what signs/symptoms I need to watch out for and what to do if I notice these signs/symptoms after I leave the hospital | € | € | € |
| I understand what I can eat, what activities and exercise I am permitted to do after I leave the hospital | € | € | € |
| I understand what medical equipment and supplies I will need after I leave the hospital, and I feel comfortable using them | € | € | € |
| **My Follow-Up** | Yes | No | Not Sure |
| My family or someone close to me knows that I am leaving and is prepared to provide the support I need. | € | € | € |
| I have a follow-up appointment scheduled with my primary healthcare provider that I am willing to keep, and I have a plan in place to get to it. | € | € | € |
| I have the name and contact information of a hospital provider I can contact if a problem arises after I leave the hospital. | € | € | € |
| I understand the tests and procedures that require follow-up, as well as the ones that I need to have after I leave the hospital. | € | € | € |
| Do you have additional concerns about your discharge?  **(Examples: anticipated discharge date, work notes, belongings, parking validation, specific questions, etc.)** | Free text | | |

**Caregiver Discharge Preparation Checklist**

| **This Patient’s Understanding** | Yes | No | Not sure |
| --- | --- | --- | --- |
| I know this patient’s main reason for being hospitalized | € | € | € |
| I know where this patient is going after s/he leaves the hospital and how s/he is getting there | € | € | € |
| My healthcare provider has answered all my questions about the plan for this patient’s care after s/he leaves the hospital. | € | € | € |
| **This Patient’s Medications** | Yes | No | Not sure |
| I understand what medications this patient will be taking (including additions and changes), what they are for, and how to take them once s/he leaves the hospital | € | € | € |
| I understand how to get this patient’s medications, and s/he will take them as prescribed after leaving the hospital | € | € | € |
| This patient will be able to cover the out-of-pocket costs for his/her medications, and his/her care team has received all authorizations required by his/her insurance plan | € | € | € |
| I understand the potential side effects of this patient’s medications and who I should contact if s/he has one | € | € | € |
| I am all set with this patient’s prescription home medications, s/he does **NOT** need any refills or renewals right now | € | € | € |
| **This Patient’s Self-Care Management** | Yes | No | Not sure |
| I understand everything I can do to keep this patient’s health problems from becoming worse | € | € | € |
| I understand what signs/symptoms I need to watch out for in this patient and what to do if I notice these signs/symptoms after s/he leaves the hospital | € | € | € |
| I understand what this patient can eat, what activities and exercise s/he is permitted to do after s/he leaves the hospital | € | € | € |
| I understand what medical equipment and supplies this patient will need after s/he leaves the hospital, and s/he feels comfortable using them | € | € | € |
| **This Patient’s Follow-Up** | Yes | No | Not sure |
| I or someone close to this patient knows that s/he is leaving and is prepared to provide the support s/he needs. | € | € | € |
| I have a follow-up appointment scheduled with this patient’s primary healthcare provider that s/he is willing to keep, and s/he has a plan in place to get to it. | € | € | € |
| I have the name and contact information of a hospital provider I can contact if a problem arises after this patient leaves the hospital. | € | € | € |
| I understand the tests and procedures that require follow-up, as well as the ones that this patient needs to have after s/he leaves the hospital. | € | € | € |
| Do you have additional concerns about this patient’s discharge?  **(Examples: anticipated discharge date, work notes, belongings, parking validation, specific questions, etc.)** | Free text | | |

**Lista de comprobación para la preparación del alta del paciente**

| **Mi entendimiento** | Sí | No | No estoy seguro(a) |
| --- | --- | --- | --- |
| Sé la razón principal por la que me hospitalizaron | € | € | € |
| Sé a dónde voy después de que salga del hospital y cómo voy a llegar allí | € | € | € |
| Mi proveedor de atención médica ha contestado todas mis preguntas sobre el plan para mi atención después de que salga del hospital | € | € | € |
| **Mis medicamentos** | Sí | No | No estoy seguro(a) |
| Entiendo cuáles medicamentos voy a tomar (incluyendo los que han añadido y los cambios), para qué sirven y cómo debo tomarlos una vez que salga del hospital | € | € | € |
| Entiendo cómo obtener mis medicamentos y los voy a tomar como me fueron recetados después de que salga del hospital | € | € | € |
| Seré capaz de cubrir los costos de mis medicamentos y mi equipo de atención médica ha recibido todas las autorizaciones requeridas por mi plan de seguro | € | € | € |
| Entiendo los posibles efectos secundarios de mis medicamentos y a quién debo contactar si tengo alguno | € | € | € |
| Ya estoy todo listo con mis medicamentos recetados para la casa y **NO** necesito renovar ninguna receta en este momento | € | € | € |
| **Mi gestión de autocuidados** | Sí | No | No estoy seguro(a) |
| Entiendo todo lo que puedo hacer para evitar que mis problemas de salud empeoren | € | € | € |
| Entiendo qué signos o síntomas necesito vigilar y qué debo hacer si observo estos signos o síntomas después de que salga del hospital | € | € | € |
| Entiendo lo que puedo comer, qué actividades y ejercicio puedo hacer después de dejar el hospital | € | € | € |
| Entiendo qué equipos y suministros médicos necesitaré después de dejar el hospital, y me siento cómodo al usarlos | € | € | € |
| **Mi seguimiento** | Sí | No | No estoy seguro(a) |
| Mi familia o alguien cercano a mí sabe que me van a dar de alta y está preparado para proporcionar el apoyo que necesito | € | € | € |
| Tengo una cita de seguimiento programada con mi proveedor de atención médica primaria que estoy dispuesto a mantener y tengo un plan de cómo llegar a la cita | € | € | € |
| Tengo el nombre y la información de contacto de un proveedor de hospital al que puedo contactar si surge un problema después de que salga del hospital | € | € | € |
| Entiendo qué pruebas y procedimientos requieren hacerle seguimiento, así como también los que necesito hacer después de que salga del hospital | € | € | € |
| ¿Tiene alguna pregunta adicional sobre su alta del hospital? (Ejemplos: fecha anticipada del alta, notas para el trabajo, pertenencias, validación de estacionamiento, preguntas específicas, etc.) |  | | |


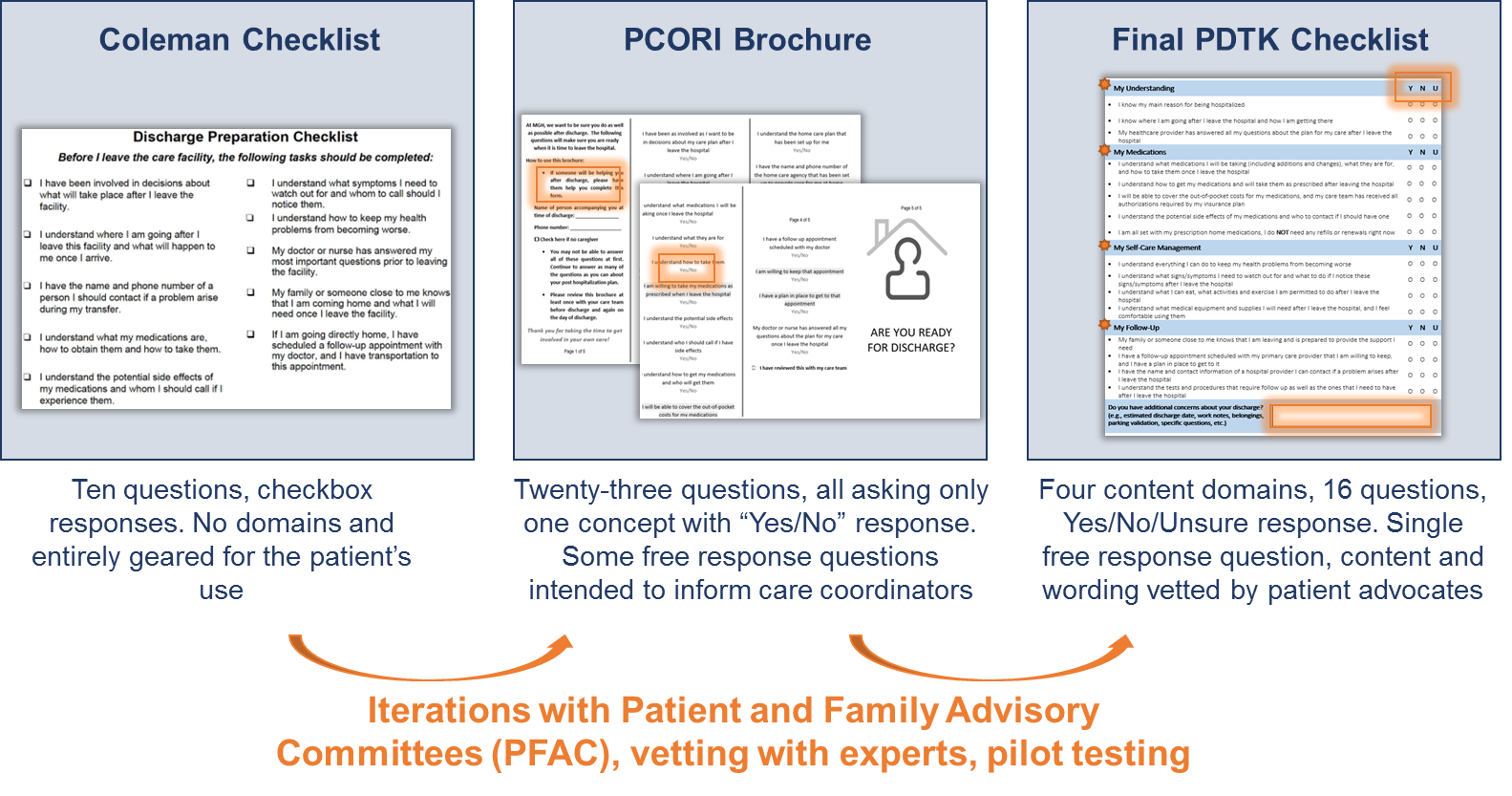

Supplement: Multimedia Appendix 2 [file jmir_v22i4e15573_app2.docx]
